# Clinical effectiveness of care managers in collaborative care for patients with depression in Swedish primary health care: a pragmatic cluster randomized controlled trial

**DOI:** 10.1186/s12875-018-0711-z

**Published:** 2018-02-09

**Authors:** Cecilia Björkelund, Irene Svenningsson, Dominique Hange, Camilla Udo, Eva-Lisa Petersson, Nashmil Ariai, Shabnam Nejati, Catrin Wessman, Carl Wikberg, Malin André, Lars Wallin, Jeanette Westman

**Affiliations:** 1 Department of Primary Health Care, Institute of Medicine, The Sahlgrenska Academy, University of Gothenburg, Gothenburg, Sweden; 2 School of Education, Health and Social Studies, Dalarna University, Falun, Sweden; 3 Region Västra Götaland, Närhälsan Research and Development Primary Health Care, Gothenburg, Sweden; 4 Health Metrics Unit, Sahlgrenska Academy, University of Gothenburg, Gothenburg, Sweden; 5 Department of Public Health and Caring Sciences - Family Medicine and Preventive Medicine, Uppsala University, Uppsala, Sweden; 6 Department of Health and Care Sciences, The Sahlgrenska Academy, University of Gothenburg, Gothenburg, Sweden; 7 Division of Nursing, Department of Neurobiology, Care Sciences and Society, Karolinska Institute, Stockholm, Sweden; 8 Division of Family Medicine, Department of Neurobiology, Care Sciences and Society, Karolinska Institute, Stockholm, Sweden; 9 Department of Primary Health Care, Institute of Medicine, The Sahlgrenska Academy, University of Gothenburg, Box 454, 40530 Göteborg, Sweden

**Keywords:** Depression, Primary care, Care manager, Collaborative care, Sick-leave, Quality-of- life

## Abstract

**Background:**

Depression is one of the leading causes of disability and affects 10-15% of the population. The majority of people with depressive symptoms seek care and are treated in primary care. Evidence internationally for high quality care supports collaborative care with a care manager. Our aim was to study clinical effectiveness of a care manager intervention in management of primary care patients with depression in Sweden.

**Methods:**

In a pragmatic cluster randomized controlled trial 23 primary care centers (PCCs), urban and rural, included patients aged ≥ 18 years with a new (< 1 month) depression diagnosis. Intervention consisted of Care management including continuous contact between care manager and patient, a structured management plan, and behavioral activation, altogether around 6-7 contacts over 12 weeks. Control condition was care as usual (CAU). Outcome measures: Depression symptoms (measured by Mongomery-Asberg depression score-self (MADRS-S) and BDI-II), quality of life (QoL) (EQ-5D), return to work and sick leave, service satisfaction, and antidepressant medication. Data were analyzed with the intention-to-treat principle.

**Results:**

One hundred ninety two patients with depression at PCCs with care managers were allocated to the intervention group, and 184 patients at control PCCs were allocated to the control group. Mean depression score measured by MADRS-S was 2.17 lower in the intervention vs. the control group (95% CI [0.56; 3.79], *p* = 0.009) at 3 months and 2.27 lower (95% CI [0.59; 3.95], *p* = 0.008) at 6 months; corresponding BDI-II scores were 1.96 lower (95% CI [− 0.19; 4.11], *p* = 0.07) in the intervention vs. control group at 6 months. Remission was significantly higher in the intervention group at 6 months (61% vs. 47%, *p* = 0.006). QoL showed a steeper increase in the intervention group at 3 months (*p* = 0.01). During the first 3 months, return to work was significantly higher in the intervention vs. the control group. Patients in the intervention group were more consistently on antidepressant medication than patients in the control group.

**Conclusions:**

Care managers for depression treatment have positive effects on depression course, return to work, remission frequency, antidepressant frequency, and quality of life compared to usual care and is valued by the patients.

**Trial registration:**

Identifier: NCT02378272. February 2, 2015. Retrospectively registered.

## Background

Depression is one of the leading causes of disability and affects 10-15% of the population [1, 2]. According to WHO, unipolar depressive disorders are the leading cause of years of healthy life lost due to disability in both men and women [2]. The majority of people with depressive symptoms seek care and are treated in primary care [1–5]. Most patients with depression have mild, moderate, or greater functional impairment that is not always congruent with the severity of the depression [6, 7]. In working life today, which is characterized by increasing high demands on cognitive performance [8], depression is one of the most common reasons for sick leave and is costly not only for the individual but also for society [9].

Depression is a common problem among patients visiting primary care. At present, best evidence internationally for high quality care and effectiveness in care of patients with depression supports collaborative care with a care manager [9].

Collaborative care interventions with care managers are organizational interventions to improve patient care by leadership support, decision support developed within the PCC, linkage to psychiatry specialist resources and community resources, and, most importantly, by engagement of the patients in their care through self-management support [10, 11]. Research shows that isolated, separate interventions are not effective for improving the treatment and management of depression in primary care [12, 13]. This means that increased waiting room screening, development of clinical guidelines, and training in refined diagnostics as separate interventions do not generate better efficiency or quality in the management and treatment of patients with depression compared to usual primary care [12, 13]. Literature reviews have shown that only those organizational measures known as collaborative care that include complex interventions can reduce depression and improve patient satisfaction and quality of life more than usual care [13–16]. Such complex interventions include measures such as education for all personnel at the primary care center (PCC) about guidelines on depression treatment and prevention; strengthening the role of nurses (care managers) who carry out telephone counseling, give treatment advice, and develop call systems; and increasing the integration between primary care and specialized care [13].

The care manager puts collaborative care into practice. Care management combines increased accessibility to the PCC via patient contacts with continuity of care for the patient and organizational and educational development at the PCC. Care management thus facilitates the care of patients with depression, improves team communication, and improves communication with secondary care (and thus continuity of care) [16]. Care managers are responsible for providing support to and maintaining continuous contact with patients with depression, training the care team, and providing feedback on the course of the patient’s depression to the physician. Studies have shown that care management is an effective strategy for successfully organizing depression treatment in primary care [13–16]. Care management increased the adequacy of antidepressant prescription, reduced patients’ symptom burden, and was cost effective [13]. However, after noting that there is a knowledge gap about care management and care managers in depression primary care treatment in Sweden, the national health care authorities called for clinical studies in Swedish primary care to evaluate the effectiveness of care management [14].

Complex interventions including organizational changes are context bound. Swedish primary care is publicly financed with salary paid GPs, organized in rather large group practices also with specialized nurses, and often including psychotherapists. Patients’ visits are fewer and longer compared to other Western countries [17].Thus, it was important to test care management in Swedish primary care to study whether it could provide more effective treatment for patients with depression than care as usual (CAU). We set out to investigate whether specially trained district nurses could facilitate effective, person-centered treatment that is concordant with evidence-based guidelines for treatment of depression in primary care.

The aim of the present study was to compare the short- and long-term effects of care management and care as usual upon remission of depressive symptoms, return to work, treatment adherence, quality of life, and patients’ satisfaction with care.

## Methods

**Fig. 1:**
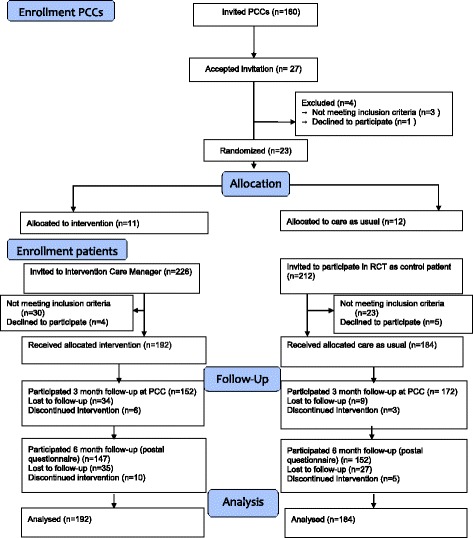
Consort flow chart of the PRIM-CARE RCT. PCCs engaged in the study and patients recruited in the study at the intervention and control PCCs from baseline and 3 and 6 months follow-up (15)

The study was designed as a pragmatic cluster randomized controlled trial of two groups (intervention and control), named the PRIM-CARE RCT (PRImary care CARE manager). The randomization was at the level of the primary care centers (PCCs). All PCCs without an onsite function at the PCC equal or comparable to a care manager in the Region Västra Götaland (VG Region) were invited to participate in the implementation of a care manager at the PCC. The implementation was designed in cooperation between the Region’s care manager implementation team and the research team, based on available evidence [7–11, 13–15], and the first wave of the implementation was carried out as the PRIM-CARE RCT. The implementation was planned to be extended to all other interested PCCs after the completion of the PRIM-CARE study. In VG Region, 160 PCCs declared an interest in participating in the implementation, and 23 of these PCCs were also interested in taking part in the research trial. Four PCCs in Region Dalarna also declared interest and were included in the study. For organizational reasons, 4 PCCs in VG Region declined participation. Consequently, the PCCs in this final group of 23 PCCs who were randomized to intervention PCCs were the first PCCs to implement a care manager function. The intervention consisted of care manager contact during 12 weeks at 11 PCCs. Control was care as usual (CAU) at 12 PCCs. Data on the PCCs are presented in Fig. 1 [18].

### Population

Patients attending 23 different urban and rural PCCs, aged ≥ 18 years, diagnosed with a new (< 1 month) mild or moderate (according to Montgomery-Åsberg Depression Rating Scale- Self assessment (MADRS-S) < 35) depression (ICD-10 diagnosis F32, F33) [19] and not diagnosed with bipolar disorder, psychosis, addiction, or cognitive impairment were included. Those not speaking/understanding Swedish were excluded. Start of inclusion was December 2014.

### Outcome

Primary outcome was patient’s depressive symptoms (measured by MADRS-S [19, 20] and BDI-II [21]) at 6 months.

Secondary outcomes were patient’s quality of life (EuroQoL-5D 3 L scale, English tariff [22]), sick leave (days), percentage return to work (RTW), antidepressant medication, and patient satisfaction (from Psychiatric Outpatient Satisfaction Scale [23]) at 3 and 6 months.

Initial data collection included age, gender, socio-demographic and economic variables, alcohol consumption, physical activity, and ethnicity. In the 3 and 6 month follow-ups, sick leave status, RTW, data on medication, comorbidity and other treatment (psychological, counseling, or other) were collected. For somatic health reasons, blood pressure and p-glucose were monitored.

### Randomization

The 23 health care centers were stratified into two strata; rural (12 health care centers) and urban (11 health care centers). Each stratum was allocated into six blocks consisting of two health care centers, in which one was randomly assigned to implement the care manager function.

### Intervention

At the intervention PCCs, a nurse devoted around 20-25% of working time as care manager for management of care for the patients with depression. Before patient recruitment began, GPs and the nurse/district nurse (care manager) participated in sessions (for GPs 2 one-day sessions and for care managers 1 three-days session before start of intervention + 2 one-day sessions during initial part of intervention) for training in providing clinical services. During the study every PCC, both intervention and control PCCs, was regularly, at least once a week, visited by the research assistants (all specialized nurses) and in addition, solely for intervention PCCs, regular follow-up meetings were held every second month where all care managers participated and where difficulties, obstacles, and successes were discussed and dealt with together with the research team and the Region’s implementation team.

Clinical services for the care manager consisted of creating an individual care plan (1 h session with patient) and further telephone contacts between nurse and patient at least 6-8 times (around 15-30 min each) during 12 weeks, with person-centered communication around depressive symptoms based on the patient’s current depression symptom assessment with a self-assessment instrument in connection with the regular telephone call, as well as behavioral activation [19, 24]. Thus, the intervention group received care as usual plus the intervention. All patients could directly contact the care manager between the scheduled telephone contacts if they needed. The care manager had direct and regular contact with the General Practitioner (GP), therapist, or other PCC personnel who were involved in the care of the patient. The care manager did not include any type of psycho-therapy in her/his care of the patient, but supported the patient and increased the accessibility and continuity of the PCC’s care for the patient, coupled with organizational changes that would facilitate care for the patient with depression (see Table 1).

**Table 1:** The care manager function in the PRIM-CARE trial: Function for the patient and function for the PCC’s organization of depression care

Care manager function for the patient	Care manger function for the PCC’s organization of depression care
Is the contact nurse for patients with depression at the PCC and facilitates the continuity and accessibility of care	Supports development of an organization for collaborative care cooperation (physician, psychologist, psychotherapist, counselor, rehabilitation personnel etc.)
Makes a structured management plan together with the patient	Facilitates cooperation with psychiatry, secondary care, community services, etc.
Keeps close cooperation with the patient’s GP and inter-professional communication	Facilitates continuity and accessibility
Follows patient symptoms by scheduled follow-ups	
Follows antidepressant treatment and possible side effects	
Pays attention to the needs of changed antidepressants or other treatment if the patient does not improve - and notifies the physician	
Provides advice on self-care and encourages behavioral activation such as planning for physical activity and pleasant events	
Informs about psychotherapy and other treatment	
Educates patients (and their families) about depression	

### Control

Participants at the control PCCs received care as usual (CAU) according to standard protocol and procedures. The Swedish National Guidelines for Depression and Anxiety Disorders recommend high accessibility and continuity, early next appointment, guided self-help, cognitive behavior therapy (CBT) (face-to-face or internet delivered), interpersonal therapy, and/or antidepressants as first and second steps in a stepped care model [3].

### Enrollment of patients and diagnostic procedure

All patients ≥ 18 years seeking care at the PCCs and who were judged to have a probable diagnosis of new (< 1 month) depression in connection with a visit to a doctor, nurse, or therapist and not judged to have any of the exclusion criteria were asked and informed about participation. The patients who accepted to participate immediately received a visit to the PCC’s care manager (intervention PCC), alternatively to a research nurse (control PCC), for diagnosis confirmation and research baseline data purposes. The depression diagnosis was confirmed by use of PRIME-MD (depression module) in accordance with DSM-IV criteria for mild/moderate depressive disorder [25].

### Statistical analysis

Standard statistical methods were used for descriptive statistics. Continuous variables were analyzed by independent sample t-test or Mann-Whitney U test and categorical variables or frequencies by Pearson chi-square test. Means of intra-individual change of depressive symptoms, and quality of life (QoL) scores were compared between the intervention group and the TAU group by using mixed model analysis with repeated measures. These analyses were adjusted for the type of PCC, age, sex, education, antidepressants at inclusion, and response variable at baseline.

The statistical analyses were made using statistical software SPSS, version 23 and SAS, version 9.4. Statistical significance was set at *p* < 0.05. No multiple adjustments were considered. We did not adjust for the cluster randomization due to sparse data in some of the health centers.

### Power calculation

The primary variable was the level of depression (as measured by MADRS-S and BDI-II) and analyzed in an ANCOVA model with repeated measures. In order to detect an effect of 3 units in the difference between the two groups, with a power of 80% and a significance level of 10% (two-sided), around 200 patients were needed in each group. The underlying assumption was a standard deviation in the group of 10 units, a within-subject correlation of 0.4, and a within-cluster correlation of 0.1, i.e. a design effect of 1.9 to correct for having a cluster analysis.

## Results

The inclusion of patients started in December 2014 and continued until January 2016, and 6 months follow-up was completed by August 2016. In all, 192 patients with mild-moderate depression (according to MADRS-S < 35) were included at the intervention PCCs with care manager and 184 patients at the control PCCs with CAU. Participation of PCCs and patients from baseline and at 3 and 6 months are shown in Fig. 1. At the 3 month follow-up, 86% participated: 79% at the intervention PCCs and 93% at control PCCs, and at 6 months 76% and 83%, respectively (postal questionnaire) (Fig. 1). Table 2 shows baseline data for intervention and control patients. There were no statistically significant differences between participants in the intervention and control patient groups at baseline concerning age, gender, lifestyle, education, occupation, sick leave, depression symptom scores (MADRS-S and BDI), or QoL.

**Table 2:** Demographics at baseline for primary care patients in the intervention group (care manager during depression) and the control group (care as usual during depression). Figures indicate numbers and percentage (%) of patients

	Intervention	Control	Total	*p*
	*n* = 192	*n* = 184	*n* = 376	
Age				
Mean (SD)	40.8 (15.0)	41.6 (15,4)	41.2 (15.2)	0.61
Gender, n(%)				
Women	131 (68.2)	137 (74.5)	268 (71.3)	0.18
Men	61 (56.0)	47 (44.0)	108 (28.7)	
BMI				
Mean (SD)	25.6 (5.57)	25.8 (5.2)	25.6 (5.6)	0.73
Occupation n (%)				
Working	137 (72.9)	122 (66.3)	259 (69.6)	
Studying	18 (9.6)	19 (10.3)	37 (9.9)	
In search of work/other	23 (17.6)	43 (23.4)	76 (20.5)	0.52
Working, n (%)				
Full-time	157 (87.7)	149 (87.6)	306 (87.7)	0.98
Other (25-75%)	22 (12.3)	21 (12.4)	43 (12.3)	
Marital status, n (%)				
Cohabiting	122 (67)	122 (68)	244 (67)	0.82
Single	61 (33)	58 (32)	119 (33)	
Born				
Outside Nordic Country n (%)	18 (9.4)	21 (11.5)	39 (10.4)	0.63
Educational level n (%)				
Primary education	17 (8.9)	27 (14.8)	44 (11.8)	
Secondary education	103 (53.9)	90 (49.2)	193 (51.9)	
University or college	71 (37.2)	66 (36.1)	137 (36.6)	0.21
Physical activity leisure time n (%)				
Sedentary	25 (13.1)	33 (17.9)	58 (15.5)	0.44
Smoking n (%)				0.26
Yes+ sometimes	45 (23.5)	56 (30.5)	101 (26.9)	
Alcohol n (%)				
once a week	14 (7.4)	14 (7.7)	28 (7.5)	0.92
Sick leave n (%)				
Sick leave last year (Yes)	83 (45.6)	66 (37.9)	149 (41.9)	0.14
On sick leave baseline	93 (50.5)	94 (55.0)	187 (52.7)	0.40
MADRS-S m (SD)	20.8 (7.2)	21.9 (7.1)	21.4 (7.1)	0.12
BDI-II m (SD)	23.9 (8.7)	25.1 (8.5)	24.5 (8.7)	0.16
EQ-5D m (SD)	0.58 (0.24)	0.56 (0.25)	0.57 (0.24)	0.41

### Non-participants

A total of 34 patients did not participate in the 3 and 6 months follow-up, 29 in the intervention group and 5 in the control group. Patients lost to follow-up were not reached despite several contacts by mail and telephone. There were statistically significant differences (*p* < 0.05) at baseline concerning age (non- participants: mean age around 10 years younger), MADRS-S (non- participants: mean value around 4 units higher) and BDI-II (non- participants: mean value around 4 units higher), and in the intervention group, a greater proportion of the non-participants were students. There were no other significant differences between participants and non-participants concerning demographic data described in Table 2.

**Fig. 2:**
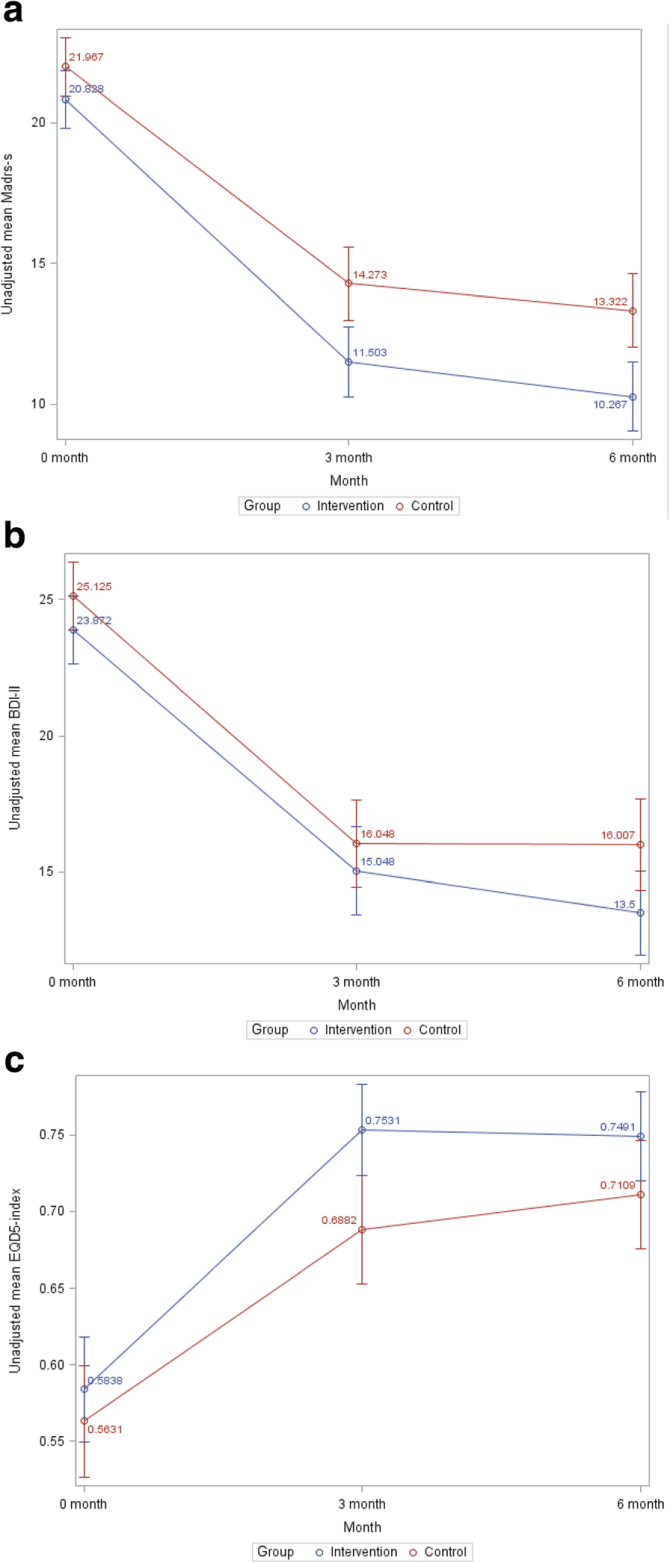
**a** Unadjusted mean of patient depression scores measured with MADRS-S at baseline, 3 and 6 months follow-up, with unadjusted confidence bars at each occasion. Statistically significant adjusted differences between intervention and control at 3 months (*p* = 0.009) and 6 months (*p* = 0.008) follow-up. **b** Unadjusted mean of patient depression scores measured with BDI-II at baseline, at 3 months (*p* = 0.67) and 6 months follow-up (*p* = 0.07) with unadjusted confidence bars at each occasion. **c** Unadjusted mean of patient quality of life scores measured with EQ-5D at baseline, at 3 months (*p* = 0.01) and 6 months follow-up (NS) with unadjusted confidence bars at each occasion

The course of depression and QoL is shown in Fig. 2a, b, and c. There was a substantial reduction of depression scores both in intervention and control groups, but the reduction was significantly greater in the intervention group compared to control group when measured with MADRS-S, and the difference still progressed during the period 4-6 months, although the care manager intervention was terminated at 3 months. Mean depression score measured by MADRS-S was 2.17 lower (95% CI [0.56; 3.79], *p* = 0.009) at 3 months and 2.27 lower (95% CI [0.59; 3.95], *p* = 0.008) at 6 months. The QoL also showed a steeper increase in the intervention group from baseline to 3 months (statistically significant difference between intervention and control at 3 months, *p* = 0.01), but this leveled off at the 6 months follow-up. Depression score reduction measured by BDI-II did not reach significance. Mean depression score measured by BDI-II was 0.44 lower (95% CI [− 1.62; 2.50], *p* = 0.67) at 3 months, and 1.96 lower (95% CI [− 0.19; 4.11], *p* = 0.07) at 6 months.

Table 3 shows remission frequency, defined as MADRS-S ≤ 12, in intervention and control group at 3 and 6 months follow-up. There was a statistically significant higher remission frequency in the intervention group at 6 months follow-up, 67% compared to 47% in the control CAU group, also illustrating the progress of depression symptom score reduction during the 4-6 months period in the intervention group.

**Table 3:** Remission frequency and use of antidepressant frequency at 3 and 6 months follow-up in the PRIM-CARE RCT. Remission defined as MADRS-S ≤ 12

	3 months Intervention N = 149	3 months Control N = 152	p	6 months Intervention N = 146	6 months Control N = 152	p
	n (%)	n (%)		n (%)	n (%)	
Remission according to MADRS-S (≤ 12)	74 (49.7)	73 (42.4)	0.20	98 (67)	72 (47)	*0.001*
Use of antidepressants	77 (50.7)	116(67.4)	*0.02*	75 (51.0)	92(60.5)	0.10
Individuals (n; %) on sick leave	83 (54.6)	88 (51.2)	0.54	59 (40.1)	64 (42.1)	0.73
Individuals (n;%) with return to work (full or part-time)	40(62.5)	33(42.9)	*0.02*	7 (33)	10 (33)	1.0

Italic figures; statistically significant difference between intervention and control group

Use of antidepressants was present at inclusion in 53% and 63% of the intervention and control group patients, respectively, and continued at the same level in the intervention group (51%), but significantly increased to 67% in the control group at 3 months follow-up (see Table 3). However, at 6 months follow-up, the antidepressant medication frequency in the control group was somewhat reduced to 61%, while the antidepressant medication frequency in the intervention group still remained stable at 51%.

**Fig. 3:**
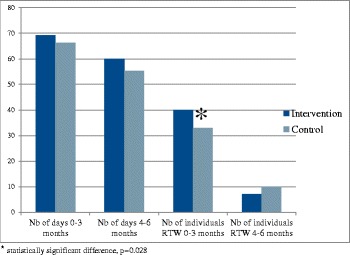
Mean number of days on sick leave from baseline to 3 months and 4 to 6 months for intervention and control group in the PRIM-CARE trial, as well as number of individuals who returned to work from baseline to 3 months, and 4 to 6 months for intervention and control group. Statistically significantly more patients in the intervention group returned via part-time sick leave to work during baseline to 3 months follow-up (62% vs 43%, *p* = 0.028)

At inclusion, 53% of the patients were on sick leave, with no significant difference between intervention and control patients (50.5% and 55%, respectively) (Fig. 3). During the period between baseline and 3 months follow-up, 55% of patients in the intervention group (83/152) and 51% (88/172) in the control group were on sick leave (ranging between 25 and 100% sick leave); mean number of sick leave days was 69.2 (intervention) and 66.2 (control). During the 4-6 months, 40% (59/147) of the intervention and 42% (64/152) of the control group patients were on sick leave for 60 and 62 mean days, respectively (Fig 3). However, significantly more patients in the intervention group returned to work during baseline to 3 months follow-up (62% vs 43%, *p* = 0.028), as significantly more patients in the intervention group returned via part-time sick leave (in Sweden 25, 50, and 75%) (Table 3, and Fig. 3).

Patients were asked by a questionnaire distributed by the research personnel (postal questionnaire at the 6 months follow-up) about the helpfulness, perception of waiting time, information given, and whether they would recommend the treatment to family members and/or close friends [23]. There were statistically significant differences between intervention and control patients concerning recommending the treatment to close friends and relatives (Table 4), but on the whole most patients were satisfied with the care given at the PCCs.

**Table 4:** Patient reported outcomes (Psychiatric Outpatient Satisfaction Scale) concerning perceived helpfulness, waiting time, information, respect of patient’s opinion, and whether the patient would recommend the treatment to family members and/or close friends

	Intervention		Control		
Opinion on	Good/excellent n (%)	Insufficient n (%)	Good/excellent n (%)	Insufficient n (%)	*p*
Helpfulness of PCC (baseline)	169 (100)	0(0)	179 (98.9)	2 (1.1)	0.17
Waiting time to visit (baseline)	165 (98.2)	3 (1.8)	165 (95.4)	8 (4.6)	0.14
Information given to you about your problem (baseline)	161 (98.2)	3 (1.8)	167 (96)	7 (4)	0.23
Information given to you about your problem (3 months)	138 (96.5)	5 (3.5)	138 91.4	13 (8.6)	0.07
Respect shown for your opinions about treatment (baseline)	163 (99.4)	1 (0.6)	163 (98.8)	2 (1.2)	0.56
Respect shown for your opinions about treatment (3 months)	130 (95.6)	6 (4.4)	140 (95.9)	6 (4.1)	0.9
Would you recommend this treatment to a friend or family member (baseline)	170 (98.8)	2 (1.2)	168 (96.0)	7 (4.0)	0.10
Would you recommend this treatment to a friend or family member (3 months)	137 (95.8)	6 (4.2)	137 (89.0)	17 (11.0)	0.03
Would you recommend this treatment to a friend or family member (6 months)	131 (97.8)	3 (2.2)	126 (86.9)	19 (13.1)	0.001

## Discussion

This study showed that PCCs that establish organizational changes concerning depression care through the implementation of a care manager improve the quality of care within a 6 month perspective, as indicated by significant reduction of depression (based on MADRS-S score), significant increase of remission frequency, QoL, and RTW compared to PCCs with care as usual for the depressed patient. Further, the patients’ antidepressant medication continued in accordance with guideline recommendations, and antidepressant medication frequency was more continuously stable when a care manager had been engaged. Patient reported satisfaction was also more favorable in PCCs with a care manager.

### Strengths and limitations

The majority of all PCCs in a region with 1.6 million inhabitants were invited to this trial. The participating PCCs, constituting around 10% of all PCCs, were representative of the region as a whole, with both urban and rural PCCs scattered over the region. Thus, the participating PCCs can be regarded as representative for Swedish primary care (total number 1200 PCCs). The number of patients recruited by the PCCs was satisfactory for attaining the pre-determined level of statistical power. The results of this trial could consequently be generalizable and representative for Swedish primary care. The attrition rate was low, and through access to the electronic patient records, complementary data especially concerning medication and sick certification were also collected.

However, there were also limitations. Due to the comprehensive organizational changes that the establishment of a care manager entailed for a PCC, concealment of the intervention status of the PCC was not possible, and all personnel at the intervention PCCs were thoroughly informed about the aim of the study and the care manager organization. The 3 months assessment at the intervention PCCs was carried out by research personnel unknown to the patient, as it could have been a possible source of bias if the assessment was made by the local care manager. At the control PCCs, the 3 months assessment was administered by a specially trained research nurse. Further, we used both MADRS-S and BDI-II as measures of depression symptom outcomes, but only MADRS-S outcomes showed statistically significant differences between the intervention and control group, although BDI-II outcomes showed similar tendencies. However, MADRS-S is an instrument specially constructed to measure change in depression course, while BDI-II is an instrument developed primarily for measuring level of depression. The instruments are complementary and show good correspondence in primary care [26], but MADRS-S is more clinically applicable for primary care and more clinically relevant concerning measurement of depression level severity [26]. Another limitation is the follow-up duration, which only covered 6 months. Important health economic consequences concerning especially care consumption, sick leave duration, and RTW for mild/medium depressed individuals should preferably be evaluated within a longer time perspective. However, in a 6 month perspective, this PRIM-CARE RCT already has shown important significant effects concerning full recovery from depression and earlier RTW, despite lower antidepressant medication frequency.

The care manager’s function at the PCC is to combine patient contacts for increased accessibility and continuity for the patient as well as facilitate support for the depressed patient through organizational changes [11]. Swedish primary care has high quality concerning medical and psycho-therapeutic competence, which is shown by the relatively modest differences between the results in the intervention and control groups. However, cornerstones of primary care such as accessibility and continuity are not sufficiently met in Swedish primary care [27], and in that respect a care manager can make a difference, especially for the group of patients with depression and anxiety, who often have low access to care due to the symptoms of the disorder. Quality improvements in Swedish primary care should also strongly enhance non-psycho-therapeutic care components to support the patient’s recovery by facilitating the patient’s own course to remission [3, 14, 15]. Further, those individuals who do not improve or deteriorate in their depression course are earlier identified by the continuous care manager contact [14].

Recently, several trials and literature reviews have been published on collaborative care with care managers in primary care [16, 28, 29]. Studies show that mental illness often negatively affects other somatic conditions, and a care manager who coordinates the care by maintaining a close and regular contact with patients and aligns efforts for their individual needs is shown not only to generate improvement of the depression but also improvement of the physical health [16]. A recent systematic review of measures in primary care leading to improved RTW also showed, similar to our study, that adding a care manager to the PCC organization is one of few factors that increase RTW for the individual [9].

The distinguishing features of a collaborative care organization with a care manager in contrast to most other types of measures already undertaken in primary care are the provision of high accessibility and continuity for the individual but also the opportunity afforded for individuals to gain knowledge about their disease, as well as the increased ability to customize care interventions to the individual’s specific needs [1, 14]. This type of care organization also allows for a better engagement of the entire PCC in terms of resources and contacts with other health care levels and society in general and provides opportunities for the continuity of these contacts. Such an organization facilitates health care that is adapted to the patient’s specific needs over time in close collaboration with the patient [14, 28, 29] and thus allows for complexity, person-centered care, and interaction on multiple levels.

## Conclusions

Our evaluation of the implementation of a care manager organization at the Swedish PCC within a 6 month perspective has shown important significant effects concerning full recovery from depression and earlier RTW for the patient, despite lower antidepressant medication frequency. The care manager organization represents a cost for the PCC in order to attain increased quality of care, especially concerning increased accessibility and continuity for the patient, and this cost should in some way be reimbursed. Further information on outcomes concerning depression, RTW, function and sick leave will be obtained in the upcoming 12 month follow-up, as well as in a health economic evaluation. The feasibility and the effectiveness of a care manager function for depressed patients also in ordinary Swedish primary care could be regarded as proven in this pragmatic RCT.

## Abbreviations

BDI-II: Beck Depression Inventory II
CAU: Care as usual
CI: Confidence interval
EQ-5D: EuroQol 5 Dimensions
GP: General Practitioner
ICD-10: International Classification of Diagnoses
MADRS-S: Montgomery-Asberg Depression Rating Scale-Self (Self-administered)
NS: Not statistically significant
PCC: Primary care centers
PRIM-CARE: PRIMary care CARE manager
QoL: Quality of Life
RCT: Randomized Controlled Trial
RTW: Return to work
TMG: Trial management group
VG Region: Region Västra Götaland
